# Domestic greywater treatment using electrocoagulation-electrooxidation process: optimisation and experimental approaches

**DOI:** 10.1038/s41598-023-42831-6

**Published:** 2023-09-22

**Authors:** Milad Mousazadeh, Nastaran Khademi, Işık Kabdaşlı, Seyedahmadreza Rezaei, Zeinab Hajalifard, Zohreh Moosakhani, Khalid Hashim

**Affiliations:** 1 Social Determinants of Health Research Center, Research Institute for Prevention of Non-Communicable Diseases, Qazvin University of Medical Sciences, Qazvin, Iran; 2 Department of Environmental Health Engineering, School of Health, Qazvin University of Medical Sciences, Qazvin, Iran; 3 Health, Safety and Environment Specialist, National Iranian Drilling Company, Ahvaz, Iran; 4 Civil Engineering Faculty, Environmental Engineering Department, İstanbul Technical University, Ayazağa Campus, 34469 Maslak, İstanbul Turkey; 5 Department of Engineering, Faculty of Civil Engineering, Persian Gulf University, Bushehr, Iran; 6 Department of Chemical Engineering, Amirkabir University of Technology, Hafez Av., Tehran, Iran; 7 Built Environment and Sustainable Technologies Research Institute (BEST), Liverpool John Moores University, Byrom Street, Liverpool, L3 3AF UK

**Keywords:** Pollution remediation, Electrochemistry

## Abstract

A synergistic combination of electrocoagulation-electrooxidation (EC-EO) process was used in the current study to treat domestic greywater. The EC process consisted of an aluminium (Al) anode and an iron (Fe) cathode, and the EO process consisted of titanium with platinum coating mesh (Ti/Pt) as an anode and stainless steel as a cathode. The effect of operative variables, namely current density, pH, EC time and EO time, on the removal of chemical oxygen demand (COD), colour, turbidity, and total organic carbon (TOC) was studied and optimised using Response Surface Methodology (RSM). The results showed that although the pH affected the removal of all studied pollutants, it had more effect on turbidity removal with a contribution of 88.44%, while the current density had the main dominant effect on colour removal with a contribution of 73.59%. It was also found that at optimal operation conditions for a current density of 2.6 A, an initial pH of 4.67, an EC time of 31.67 min, and an EO time of 93.28 min led to a COD, colour, turbidity, and TOC removal rates of 96.1%, 97.5%, 90.9%, and 98%, respectively, which were close to the predicted results. The average operating cost and energy consumption for the removal of COD, colour, turbidity, and TOC were 0.014 $/m^3^ and 0.01 kWh/kg, 0.083 $/m^3^ and 0.008 kWh/kg, 0.075 $/m^3^ and 0.062 kWh/kg, and 0.105 $/m^3^ and 0.079 kWh/kg, respectively.

## Introduction

Water has played an important and diverse role in human life and civilisation, so water-related issues such as scarcity and poor water quality must be addressed^1^. One of the most successful solutions to this problem is the management of non-renewable water resources, especially in developing countries. Greywater (GW) reuse is an essential water conservation approach^2^. GW is a fraction of domestic wastewater from showers, handwashing basins, laundry, and kitchen sinks, whereas black water includes wastewater from toilets and bidets. GW accounts for 50–75% of water usage; hence, reusing GW can be an excellent technique for addressing water scarcity. GW contains fat, oil, numerous anions and cations, other organic compounds from cooking, and residues from daily personal care products such as toothpaste, shampoo, soap, shower gel, and detergent surfactants. Toxic micropollutants, such as those from personal care and home items, are also found in GW^3^.

Most treatment methods for removing total suspended solids (TSS) and organic matter have opted to meet the regulations and needs of GW reuse, such as irrigation and toilet flushing. Coagulation/flocculation^4^ or biological treatment processes such as constructed wetland^5^, rotating biological contactor (RBC)^6^, sequencing batch reactor (SBR)^7^, membrane bioreactor (MBR)^8^, and up-flow anaerobic sludge reactor (UASB)^9^ have been proved not to offer the full treatment of GW. It should also be noted that each of these processes has their own drawbacks. For instance, constructed wetlands have low selectivity and need a large surface area; regeneration of adsorbents is a crucial factor in adsorption processes, and membrane filtration technologies usually require high operating costs^10^. On the other hand, advanced oxidation processes (AOPs) have been widely used in recent years to treat various effluents and have been proven to be fruitful. According to findings published in the scientific literature, AOPs can improve industrial wastewater biodegradability, eliminate micropollutants from polluted water, and offer high-quality water for reuse^11^. To limit the risk of human contact, GW reuse for agriculture activities requires advanced treatment. Several advanced oxidation processes, such as Fenton oxidation^12^, persulfate oxidation^13^, photocatalysis^14^, photooxidation^15^, photo-Fenton^16^, and ozonation^17,18^, have been studied for this purpose.

**Figure 1:**
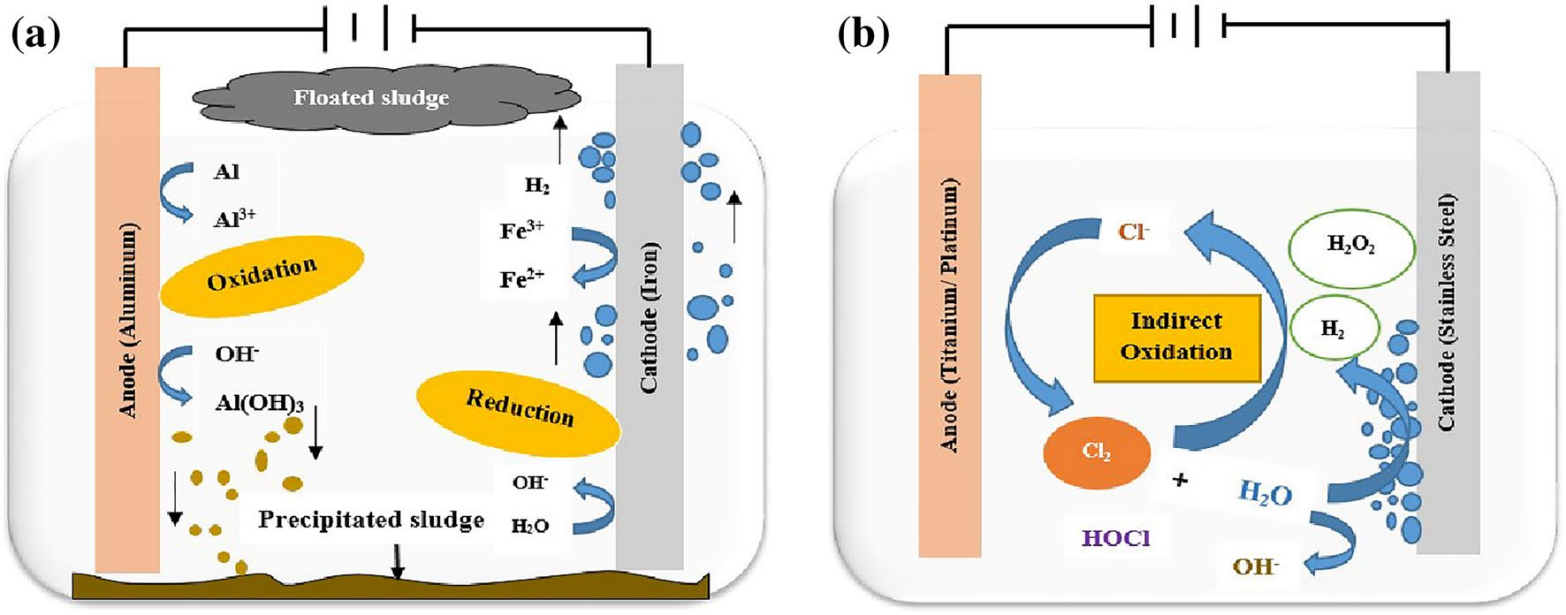
Principal reactions in (**a**) electrocoagulation and (**b**) electrooxidation process.

During the last decade, special attention was paid to hybrid processes for treating different effluents. Among them, electrocoagulation (EC) successfully removes several organic and inorganic matter from wastewater^19–21^. When it comes to treating a wide range of wastewater, EC has shown to be a flexible method^22^. Agglomeration of contaminants is accomplished via the EC method, which employs electrochemically created iron and aluminium coagulants (Fig. 1a)^23^. The oxidation of anodes produces cations (Fe^3+^ and Al^3+^) that can destabilise colloidal systems^24^. Recently, AOPs have been coupled by this process to create free radicals for the oxidation of organic molecules to increase the performance of the EC process. Even yet, it is true that the EC/AOPs technique can be applied to separate and destroy a wide range of contaminants^25^.

Electrooxidation (EO) oxidises pollutants by forming hydroxyl radicals on the anode or creating oxidants in the solution^26^. Its performance highly depends on the anode material^27^. Anodic oxidation can be indirect or direct based on the responsible reaction mechanism. However, partial oxidation occurs in indirect oxidation, while full mineralisation occurs in direct oxidation^28^. During direct oxidation, also called electrochemical oxygen transfer reaction, after adsorbing onto the anode surface, direct electron transport oxidises pollutants on the anode. In contrast, during indirect electrooxidation, also termed mediated anodic oxidation, chemical oxidants are produced in situ via anodic oxidation, such as active chlorine and persulphate or via cathodic reduction, such as hydrogen peroxide. It is also possible to produce active chlorine species by electrooxidation in the presence of chloride ions (Fig. 1b). The production pathways of hydroxyl radicals and active chlorine species are given in Eqs. (1), (2), (3) and (4)^29^.

1 $${\text{M }} + {\text{ H}}_{2} {\text{O}} \to {\text{M}}({}^{ \cdot }{\text{OH}}) \, + {\text{ H}}^{ + } + {\text{ e}}^{ - }$$

2 $${\text{2H}}_{{2}} {\text{O}} \to {\text{O}}_{{2}} + {\text{ 4H}}^{ + } + {\text{ 4e}}^{ - }$$

3 $${\text{2Cl}}^{ - } \to {\text{Cl}}_{{2}} + {\text{ 2e}}^{ - }$$

4 $${\text{Cl}}_{{2}} + {\text{ H}}_{{2}} {\text{O}} \to {\text{ClOH }} + {\text{ Cl}}^{ - } + {\text{ H}}^{ + }$$

Compared to other advanced oxidation processes, EO offers several advantages, such as eliminating the need for chemicals, the small footprint, and the reduction of pathogens^30^. Various electrodes coated with metal oxides have been examined for their electrocatalytic activity in the EO process. These anodic electrodes include graphite, Ti/Pt, Ti/Pt-Ir, Ti/PbO_2_, Ti/PdO-Co_3_O_4_ and Ti/RhOx-TiO_2_, Ti coated with oxides of Ru/Ir/Ta and BDD^31^. Ghimire et al.^32^ investigated using mesh-type platinum-coated titanium (Ti/Pt) to reduce COD and ammonium in domestic wastewater. They found that the indirect electrooxidation of pollutants proceeded well on a Ti/Pt anode, which achieved over 97% removal efficiency. In addition to enhancing the process, various anodically-generated oxidising agents such as peroxide, Fenton’s reagent, sodium chloride, chlorine, hypochlorite, or peroxodisulfate were added to the wastewater to react with both organic and inorganic pollutants.

Barisci et al.^33^ compared eight combinations of electrodes in treating domestic GW by EC and found that the highest COD removal was attained with the Al–Fe–Fe–Al combination at a current density of 1 mA/cm^2^ and an initial pH of 7.62. These operation conditions yielded almost complete anionic surfactant (MBAS) removal, while COD concentration reduced from 229 to 4.4 mg/L. No noticeable improvement in process performance was observed at the highest current density tested (1.5 mA/cm^2^). Other studies have also shown the practicality of EC for GW treatment^34^.

In contrast to EC, EO is a more time-consuming process but reliably eliminates pollutants^35^. Low or no chemical requirements and ease of application make EC and EO the most promising electrochemical treatment methods^36^. Rubí-Juárez et al.^37^ treated a carwash wastewater, the character of which was very close to GW, by the integrated EC-EO process using Al and boron-doped diamond (BDD) electrodes and reached 82% COD removal.

To the best of the author’s knowledge, there is no scientific research in which the combination of these processes is used to treat real GW, and the only EC-AOP process used in this area was the EC-Ozone combination^17,18^. The treatment of GW by the hybrid EC-EO process using an Al anode and iron cathode in EC and Ti/Pt anode and stainless steel cathode in EO was proposed for the first time, which also overcomes the relatively high operating costs of the AOPs. This process was previously used for dairy wastewater treatment and proved effective as Ti/Pt is a stable material for electrooxidation^38^.

According to the literature^39^, EO has successfully removed pollutants from water containing soluble organic matter. However, it is unsuitable for purifying water with a high suspended solids concentration. As a result, before directing wastewater to an EO treatment system, it is necessary to treat it first using a proper method to eliminate the suspended solids. Therefore, this study combined the EO system with the EC method to effectively remove COD, colour, turbidity, and TOC from GW. Additionally, Response Surface Methodology (RSM) and Central Composite Design (CCD) were used to optimise the effect of key variables on the performance of the EC-EO hybrid process in terms of turbidity, colour, and organic matter (COD and TOC) removal. The optimised variables were the current density, initial pH and electrolysis time.

## Materials and methods

### GW characterisation

The required samples of GW were collected from residential units (generated from bathtubs, showers, hand basins, kitchen sinks, and laundry room sinks) in Qazvin, Iran, using polypropylene containers and stored at 4 °C in the laboratory. Characterisation of these GW samples is outlined in Table 1, which shows that the GW samples have high strength compared to the published data, the COD concentrations varied between 1300 and 2000 mg/L (average 1560 mg/L), and intense colour (850–2000 ADMI) was observed. The turbidity of the samples ranged from 150 to 500 NTU, which is attributed to high particulate matter content (particularly COD and particulate matter).

**Table 1:** The characteristics of raw GW used in the present study.

Parameters	Unit	Present study		^40^		^41^	^33^
		Min	Max	Min	Max		Average
pH	–	8.9	9.7	5.2	10.2	5.78	7.62 ± 0.032
Conductivity	mS/cm	3.8	5	0.36	1.3	–	802.2 ± 0.073*
TOC	mg/L	70	260	–	–	–	–
COD	mg/L	1300	2000	40	1270	646	229 ± 3.21
Colour	ADMI	850	2000	155	560	–	–
Turbidity	NTU	150	500	26	318	–	53.4 ± 1.12

*μS/cm.

### Analytical methods and instruments

All analyses were performed following the Standard Methods^42^. COD was measured according to 5220 D: Closed Reflux, Colourimetric Method using a digestion reactor (LT200, Hach, USA) and direct reading spectrophotometer (DR 6000: UV–Vis, Hach, Germany) at a wavelength of 620 nm. After acidification, the TOC content of samples was determined following the 5310B: High-Temperature Combustion Method using a multi-parameter TOC analyser (CONSORT C831, Belgium). Turbidity and colour measurements were conducted according to 2130 B: Nephelometric Method and 2120 F: ADMI Weighted-Ordinate Spectrophotometric Method, using a spectrophotometer (2100 AN, Hach, Germany) and a spectrophotometer (DR 6000 UV–Vis, Hach, Germany), respectively. A digitally calibrated conductometer (Leybold 666,222, Germany) and a multi-parameter analyser (CONSORT C831, Belgium) were used to measure the electrical conductivity and pH of the samples, respectively. The removal of GW was calculated using the following equation^43^:

5 $${\mathrm{R}}_{\mathrm{e}}\left(\mathrm{\%}\right)=\frac{\left({\mathrm{C}}_{0}-{\mathrm{C}}_{\mathrm{t}}\right)}{{\mathrm{C}}_{0}}\times 100$$

where C_0_ and C_t_ are the initial and final concentrations of the pollutants, respectively.

### Experimental set-up of batch sequential EC-EO and procedure

**Figure 2:**
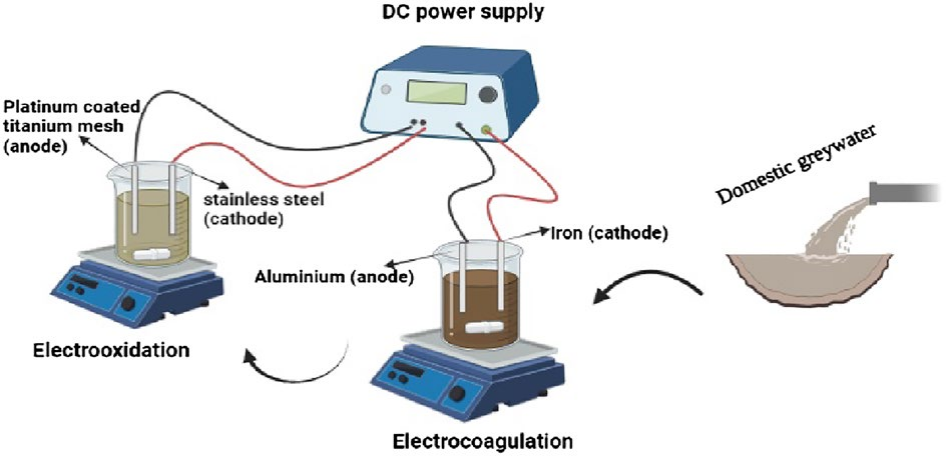
Experimental set-up used in the present study.

Figure 2 depicts the experimental set-up. In the EC reactor, the Al anode and Fe cathode were connected to a direct current power supply (model JPS303D, Iran) to supply adjustable voltage (0–30 V) and applied current (0–3 A) in a monopolar arrangement. EC electrodes had a surface area of 40 cm^2^ and an inter-electrode spacing of 1 cm. A magnetic stirrer (model SHA R-50, Iran) was used to ensure the complete mixing of the fluid throughout the EC cell. In each run, 550 mL of GW was fed into the EC reactor after pH adjustment using H_2_SO_4_ or NaOH (1 M). The effluent of EC then flowed through the EO cell, which has a similar configuration to the EC unit with mesh-type platinum-coated titanium (Ti/Pt) with the dimensions of 14 × 10 × 1 mm as anode and flat-plate stainless steel (grade 304) of the same size as a cathode. The material used for the anode had a platinum coating of 1 µm thickness. It was produced through a series of processes, including punching, pulling, annealing, acid-washing, and coating.

After each run, a sample of 20 mL was drawn with a syringe from the centre of the EO reactor at specified time intervals. The effluent samples were analysed after centrifuging at 3000 rpm for 15 min. EC electrodes were cleaned completely with sandpaper and rinsed with water at the end of each run to eliminate any solid residues on the surfaces to avoid passivation.

### Response surface methodology

RSM and CCD are effective statistical tools for modelling and optimising the simultaneous impacts of key parameters within a four-factor, five-level framework^44,45^. These five levels are categorised as two axial points, two factorial points, and one central point for each variable. These two statistical tools were used in this study for the modelling and optimising the effects of four key variables, namely current density (0.75–3.75 A), initial pH (2–12), EC time (10–50 min), and EO time (15–95 min), on the performance of the EC-EO process in terms of COD, colour, turbidity, and TOC removal efficiency. The four variables are coded as shown in Table 2, noting that -α and + α represent the extreme levels for each variable.

**Table 2:** The coded levels and range of the studied variables.

Variables	Unit	Type	Coded levels				
			− α	− 1	0	+ 1	+ α
Current density	A	A	0.75	1.5	2.25	3	3.75
Initial pH	–	B	2	4.5	7	9.5	12
EC time	min	C	10	20	30	40	50
EO time	min	D	15	35	55	75	95

Factorial designs only predict the variables’ linear trend and cannot determine curvature or critical points in the designed space^46^; therefore, RSM was used to model the process with the least number of experimental runs and with a precise analysis of the variables in different formats. Based on the number of variables, a specific number of experiments are designed according to the following equation:

6 $$E = 2^{v} + 2V + P$$

where *E* is the number of demanded experiments for analysis, *V* is the number of independent variables, and *P* is the number of replications at the central point. Twenty-nine trials were designed using parameters from a framework of four variables and five stages in the present study. The empirical model, as shown by Eq. (7), was a second-order polynomial regression, which was used to study the interaction between y and the independent variables:

7 $$y={\beta }_{0}+\sum_{i=1}^{k}{\beta }_{i}{x}_{i}+\sum_{i=1}^{k}{\beta }_{ii}{x}_{i}^{2}+\sum_{1\le i\le j}^{k}{\beta }_{ij}{x}_{i}{x}_{j}+\varepsilon$$

where *y* denotes the result (removal efficiency of COD, TOC, turbidity, and colour in percentage), *j* is second-order, *i* is the linear constant, *β*_*0*_ is a constant coefficient, *β*_i_ is the regression constant, *β*_*ii*_ is the quadratic coefficient, and *β*_*ij*_ is the interaction coefficient. Also, *x*_*i*_ and *x*_*j*_ are the coded independent variables.

## Result and discussion

### RSM experimental design

After using the five-level coding scheme shown in Table 3, the CCD method was performed to assess the effect of the studied variables on COD, TOC, turbidity and colour removal by the EC-EO process. The number of replications at the central points for each variable is to identify the errors of the model and the quality of the final regression. Among 29 experimental runs, 24 runs were designed in factorial and axial points, while the rest of the runs (#4, #6, #12, #16, and #19) were at the central point (2.25 A, 7, 30 min, and 55 min) replicated.

**Table 3:** CCD experimental design and the response results for GW treatment using EC-EO processes.

Run	A (A)	B	C (min)	D (min)	Response 1 COD removal (%)		Response 2 colour removal (%)		Response 3 turbidity removal (%)		Response 4 TOC removal (%)	
					Predicted	Actual	Predicted	Actual	Predicted	Actual	Predicted	Actual
1	2.25	7	30	15	92.61	92.38	93.51	93.17	88.64	90.33	90.85	91.31
2	1.5	4.5	40	75	92.80	93.61	87.74	87.64	96.07	94.95	97.94	98.7
3	1.5	4.5	20	35	86.01	87.30	84.29	84.35	99.06	99.73	94.36	95.14
4	2.25	7	30	55	93.66	93.53	95.67	95.88	93.65	93.99	95.32	95.57
5	3	4.5	20	75	95.54	97.84	98.89	98.35	96.40	96.66	94.78	94.46
6	2.25	7	30	55	93.66	93.84	95.67	94.70	93.65	92.17	95.32	94.17
7	3	9.5	40	35	88.61	89.15	99.56	98.58	80.10	79	82.59	82.85
8	3	9.5	20	35	92.53	92.81	95.93	96.35	76.13	76.33	81.48	81.29
9	1.5	4.5	20	75	91.71	91.56	88.40	89.16	98.51	98.99	99.31	99.39
10	1.5	9.5	20	75	90.39	90.68	86.84	87.05	79.51	78.12	93.23	93.63
11	1.5	9.5	20	35	89.52	89.69	84.74	85.27	77.72	76.12	91.02	90.49
12	2.25	7	30	55	93.66	93.12	95.67	95.22	93.65	93.37	95.32	95.59
13	3	4.5	40	35	90.67	91.87	96.57	96.58	97.51	97.6	89.64	89.81
14	3.75	7	30	55	100.85	99.99	96.24	96.30	90.55	90.73	87.75	87.87
15	0.75	7	30	55	92.83	92.68	74.29	73.16	90.77	92.19	99.78	98.71
16	2.25	7	30	55	93.66	94.75	95.67	96.25	93.65	95.16	95.32	96.07
17	3	9.5	40	75	90.93	90.75	99.48	99.45	82.36	80.90	85.66	85.55
18	1.5	9.5	40	35	84.43	83.35	88.09	88.65	78.21	77.09	90.10	90.99
19	2.25	7	30	55	93.66	92.80	95.67	95.55	93.65	93.49	95.32	95.19
20	1.5	4.5	40	35	77.36	77.65	83.04	82.35	96.97	96.21	92.31	91.91
21	3	9.5	20	75	85.10	85.15	95.27	95.5	78.74	78.83	83.86	84.67
22	2.25	12	30	55	73.82	74.36	94.79	94.35	63.86	65.97	76.32	75.36
23	3	4.5	20	35	98.15	97.64	97.55	97.90	96.13	95.23	89.65	88.72
24	2.25	7	50	55	91.28	91	95.53	96.55	91.17	92.5	91.96	90.51
25	1.5	9.5	40	75	95.05	95.5	90.79	90.25	79.65	79.89	92.98	94.28
26	2.25	7	10	55	94.10	93	92.56	91.36	89.64	89.92	92.21	92.63
27	3	4.5	40	75	97.81	97.5	98.50	97.92	97.44	98.4	95.46	96.4
28	2.25	7	30	95	100.62	99.78	97.55	97.78	90.35	90.35	98.87	97.43
29	2.25	2	30	55	77.19	75.13	93.36	93.78	100.28	99.78	89.45	89.42

### Development and validation of RSM models

The derived model and its parameters were evaluated using the analysis of Variance (ANOVA) technique. Table 4 presents the results, which are used in ANOVA calculations. These calculations involve taking data from each experimental run and using it to determine the terms in the mathematical model. The average and variance of each term are then calculated, along with the mean of the variances of that term. This is done by dividing the sum of the variances of the samples by the degree of freedom (*df*) of the corresponding term. It can be said that *df* is defined as the number of parameters used to define the related term^47^. The mathematical model predicts each response with a specific set of experimental conditions. This prediction is slightly different from the experimental results. The difference between the experimental and mathematical results is defined as the residue of those data. The significance of each term is explained as the ratio of the mean variances of that term to the mean variances of the residuals. The resulting ratio is called the “*F-*value” of that term, demonstrating the model’s potency in predicting responses in a wide range of operational conditions. The *F-*value of a model must be more significant than the *p*-value of the model to acquire the correct results. The COD, colour, turbidity and TOC model F values were calculated as 61, 104.6, 78.17, and 55.97, respectively. Lack of fit (LOF) indicates the weakness of the model or the proportion of the errors resulting from the mathematical inaccuracy of the derived equation. LOF is calculated by replicating limited experiments in the central point discussed in the previous sections. It is anticipated that the *F-*value of LOF is insignificant^48^. In the present study, the estimated LOF value for the model was found to be insignificant for all responses (*p*-value > 0.05), namely the COD, colour, turbidity and TOC (*p*-values: 0.1577, 0.2356, 0.2959, and 0.1745; respectively). Therefore, it could be said that the model is accepted, suitable, and considerable for pollutant removal using the EC-EO hybrid process. The obtained result shows the deviation between the experimental and predicted data; the residues are due to random errors, which should constitute a normal distribution^49^. “P-value” is the probability of the hypothesis that an important breakthrough occurs in the model accuracy while adding a specific term, and it is another parameter that demonstrates the significance of the model and the parameter^50^. It was also found that the P-value was less than 0.0001 for all four responses, confirming that the model was correct and highly significant.

**Table 4:** ANOVA results of quadratic for COD, colour, turbidity, and TOC removals.

Source of variations	Sum of square				Degree of freedom				Mean square				F-value				P-value pro. > F			
	R_COD_	R_Colour_	R_Turbidity_	R_TOC_	R_COD_	R_Colour_	R_Turbidity_	R_TOC_	R_COD_	R_Colour_	R_Turbidity_	R_TOC_	R_COD_	R_Colour_	R_Turbidity_	R_TOC_	R_COD_	R_Colour_	R_Turbidity_	R_TOC_
Model	1164.61	982.53	2249.44	869.20	14	14	14	14	83.19	70.18	160.67	62.09	61.00	104.65	78.17	55.97	< 0.0001*	< 0.0001*	< 0.0001*	< 0.0001*
A-current density	96.52	723.03	0.07	217.08	1	1	1	1	96.52	723.03	0.07	217.08	70.78	1078.16	0.034	195.70	< 0.0001*	< 0.0001*	0.8558	< 0.0001*
B-pH	17.05	3.07	1989.62	258.60	1	1	1	1	17.05	3.07	1989.62	258.60	12.50	4.58	967.98	233.12	0.0033^	0.0503	< 0.0001*	< 0.0001*
C- EC time	11.97	13.22	3.50	0.09	1	1	1	1	11.97	13.22	3.50	0.097	8.78	19.71	1.70	0.09	0.0103^	0.0006^	0.2132	0.7726
D- EO time	96.28	24.42	4.40	96.40	1	1	1	1	96.28	24.42	4.40	96.40	70.60	36.42	2.14	86.91	< 0.0001*	< 0.0001*	0.1654	< 0.0001*
AB	83.31	4.25	1.80	23.38	1	1	1	1	83.31	4.25	1.80	23.38	61.09	6.34	0.87	2.14	< 0.0001*	0.0246^	0.3658	0.0004^
AC	1.36	0.07	12.08	4.18	1	1	1	1	1.36	0.07	12.08	4.18	0.99	0.10	5.87	0.87	0.3344	0.7488	0.0295^	0.0726
AD	68.93	7.66	0.67	0.03	1	1	1	1	68.93	7.66	0.67	0.03	50.55	11.42	0.33	5.87	< 0.0001*	0.0045^	0.5764	0.8631
BC	12.69	21.14	6.68	1.27	1	1	1	1	12.69	21.14	6.68	1.27	9.31	31.52	3.25	0.32	0.0086^	< 0.0001*	0.0929	0.3035
BD	23.30	4.03	5.48	7.54	1	1	1	1	23.30	4.03	5.48	7.54	17.09	6.01	2.66	3.25	0.0010^	0.0280^	0.1249	0.0207^
CD	95.01	0.35	0.12	0.47	1	1	1	1	95.01	0.35	0.12	0.47	69.67	0.52	0.058	2.66	< 0.0001*	0.4813	0.8133	0.5260
A^2^	16.35	175.66	14.51	3.95	1	1	1	1	16.35	175.66	14.51	3.95	0.04	261.93	7.06	0.06	0.0038^	< 0.0001*	0.0188^	0.0801
B^2^	534.75	4.17	217.32	250.97	1	1	1	1	534.75	4.17	217.32	250.97	392.13	6.21	105.73	7.06	< 0.0001*	0.0258^	< 0.0001*	< 0.0001*
C^2^	1.54	4.30	17.04	16.97	1	1	1	1	1.54	4.30	17.04	16.97	1.13	6.41	8.29	105.73	0.3060	0.0240^	0.0121^	0.0016^
D^2^	14.12	0.03	28.01	0.34	1	1	1	1	14.12	0.03	28.01	0.34	10.35	0.04	13.63	8.29	0.0062^	0.8275	0.0024^	0.5866
Residual	19.09	0.03	28.78	15.53	14	14	14	14	1.36	0.67	2.06	1.11								
Lack of fit	16.78	7.93	23.59	13.53	10	10	10	10	1.68	0.79	2.36	1.35	2.91	0.79	1.82	13.63	0.1577	0.2356	0.2959	0.1745
Pure error	2.31	1.46	5.18	2.00	4	4	4	4	0.58	0.36	1.30	0.49		0.36						
Cor Total	1183.70	991.92	2278.21	884.73	28	28	28	28												
R_COD_	R^2^/R^2^_adj_ (%) = 0.98/0.97			C.V. % = 1.28																
R_Colour_	R^2^/R^2^_adj_ (%) = 0.94/0.95			C.V. % = 0.88																
R_Turbidity_	R^2^/R^2^_adj_ (%) = 0.94/0.88			C.V. % = 1.15																
R_TOC_	R^2^/R^2^_adj_ (%) = 0.98/0.96			C.V. % = 1.61																

*Highly significant, ^Significant.

The results shown in Table 4 proved that the developed quadratic model was statistically significant as it has 99% confidence and a p-value of 0.0001 for removing all modelled pollutant parameters. Similarly, all factors have substantial effects on COD removal (ANOVA, *p-value* < 0.05) except C^2^ (*p-value* = 0.3060) and interaction of AC (*p-value* = 0.3344). For the turbidity removal, all quadratic coefficients (A^2^, B^2^, C^2^, and D^2^), linear effect of B, and interaction of AC were significant, while the other factors were insignificant. The high values of R^2^ (0.98) and Adj-R^2^ value (0.97), the coefficient of variation (C.V.) value (1.15%) and the insignificant value for LOF (23.59) confirmed that the mathematical model is applicable to the turbidity removal according to the various combination of variables values. All interactions except AB and quadratic coefficients of A^2^ and B^2^ were insignificant for TOC removal. All linear effects (A, B, C and D) have a substantial impact on TOC removal (except C) and colour removal (except B). Relatively high values of R^2^ verified the applicability of the quadratic model developed for both colour and TOC removals from GW by the EC-EO hybrid process.

The predicted vs. actual plot in Design Expert software shows R^2^ in graphical form; see Fig. S1. Experimental data are anticipated to be distributed evenly and with the minimum deviation from the predicted data in the R^2^ graph. Fig. S1 clearly shows that all the responses and experimental data are well distributed on the y = x line, indicating the accuracy of the regression and the polynomial model.

The residuals are anticipated to be mostly from random errors, which should be distributed normally. As seen in Fig. S2, the residuals are distributed semi-evenly around the normal line, which indicates the prediction potency of the model.

RSM could not derive cubic or higher exponent equations due to the limited number of tests. However, it is capable of modelling data in a quadratic equation. For all four responses, the quadratic equation with coded coefficients (− α, − 1, 0, 1, + α) for each term is demonstrated in Eqs. (8) to (11). The coded equations are proportional to the real states in their coefficient; however, the coefficients are scaled to the dimensions that the variables are analysed in a three-dimensional space. In the equation, the bigger the absolute value of a term, the more impact it makes on the response. Also, the negative coefficients represent the adverse effect on the response.

8 $$ \begin{aligned}{\text{COD removal }}\left( \% \right) \, & = \, + {93}.{66 } + { 2}.0{\text{1 A }} - \, 0.{\text{84 B }} - \, 0.{\text{71 C }} + { 2}.00{\text{ D }} - { 2}.{\text{28 AB }} + \, 0.{\text{29 AC }} - { 2}.0{\text{8 AD }} \\ &\quad+ \, 0.{\text{89 BC }} - {1}.{\text{21 BD }} + { 2}.{\text{44 CD }} + \, 0.{\text{79 A}}^{2} \, - { 4}.{\text{54 B}}^{2} \, - \, 0.{\text{24 C}}^{2} \, + \, 0.{\text{74 D}}^{2}\end{aligned} $$

9 $$ \begin{aligned}{\text{Colour removal }}\left( \% \right) \, & = \, + {95}.{67 } + { 5}.{\text{49 A }} + \, 0.{\text{36 B }} + \, 0.{\text{74 C }} + { 1}.0{\text{1 D }} - \, 0.{\text{52 AB }} + \, 0.0{\text{7 AC }} - \, 0.{\text{69 AD }} \\ &\quad+ { 1}.{\text{15 BC }} - \, 0.{5}0{\text{ BD }} + \, 0.{\text{15 CD }} - {2}.{6}0{\text{ A}}^{{2}} - \, 0.{4}0{\text{ B}}^{{2}} - \, 0.{\text{41 C}}^{2} - \, 0.0{\text{4 D}}^{{2}}\end{aligned} $$

10 $$ \begin{aligned}{\text{TOC removal }}\left( \% \right) \, & = \, + {95}.{322 } - { 3}.0{\text{1 A }} - { 3}.{\text{28 B }} - \, 0.0{\text{6 C }} + { 2}.00{\text{ D }} - { 1}.{\text{21 AB }} + \, 0.{\text{51 AC }} + \, 0.0{\text{5 AD }} \\ &\quad+ \, 0.{\text{28 BC }} - \, 0.{\text{69 BD }} + \, 0.{\text{17 CD }} - \, 0.{\text{39 A}}^{{2}} - { 3}.{\text{11 B}}^{{2}} - \, 0.{\text{81 C}}^{{2}} - \, 0.{\text{11 D}}^{{2}}\end{aligned} $$

11 $$ \begin{aligned}{\text{Turbidity removal }}\left( \% \right) \, & = \, + {93}.{648 } - \, 0.0{\text{5 A }} - { 9}.{1}0{\text{ B }} + \, 0.{\text{38 C }} + \, 0.{\text{43 D }} + \, 0.{\text{33 AB }} + \, 0.{\text{87 AC }} \\ & \quad+ \, 0.{2}0{\text{ AD }} + \, 0.{\text{65 BC }} + \, 0.{\text{58 BD }} - \, 0.0{\text{9 CD }} - \, 0.{\text{75 A}}^{{2}} \\ &\quad- { 2}.{\text{89 B}}^{{2}} - \, 0.{\text{81 C}}^{{2}} - {\text{ 1.04 D}}^{{2}}\end{aligned} $$

### Model analysis via 3-D surface 

#### Effect of current density and initial pH on the process performance

Electrochemically produced Al^3+^ undergoes hydrolysis in the bulk solution to form hydrolysis products, which involve charge neutralisation and sweep flocculation of colloids or adsorption of pollutants onto Al hydroxide during EC process^51^. Increasing initial pH and current density speed up these hydrolysis-polymerisation reactions. ${\text{Al}}{{\text{(H}}_{2}\text{O)}}_{4}{\text{(OH)}}^{2+}$, ${\text{Al}}{{(\mathrm{H}}_{2}\mathrm{O})}_{5}{(\mathrm{OH})}^{2+}$, ${\text{Al}}{(\mathrm{OH})}^{2+},$ ${\text{Al}}{\text{(OH)}}_{2}^{+}$, ${\text{Al}}{(\mathrm{OH})}_{4}^{-}$, ${\text{Al}}_{2}{\text{(OH)}}_{2}^{4+}$, ${\text{Al}}_{6}{\text{(OH)}}_{15}^{3+}$, ${\text{Al}}_{7}{\text{(OH)}}_{17}^{4+}$, ${\text{Al}}_{8}{\text{(OH)}}_{20}^{4+}$, ${\text{Al}}_{13}{\text{(OH)}}_{34}^{5+}$, and ${\text{Al}}_{13}{{\text{O}}}_{4}{\text{(OH)}}_{24}^{7+}$ can form through these reactions depending on the solution pH^51–54^. In the pH range of 4–7, the positively charged polymeric Al hydroxo complex species dominate in the reaction solution. These species are important in destabilising negatively charged particles through charge neutralisation. Among the polymeric species, Al_13_ is the dominant species in situ formed during 5–15 min of EC time in the pH range of 5–7 at low current intensities^51^. Al_13_ is more readily available for adsorption and charge neutralisation at around neutral pH^55^. Amorphous Al(OH)_3_(H_2_O)_3_ forms in the pH range of 5–8, providing a larger surface area for rapid adsorption of soluble organic compounds and trapping of the colloidal pollutants^56^. At alkaline pH values (> 9), this destabilisation capacity reduces as negatively charged ${\text{Al}}{\text{(OH)}}_{4}^{-}$ becomes dominant species^51^, or the number of positive surface sites of freshly formed Al(OH)_3_ to be attached decreases at a pH higher than pH_zpc_ of 8.4^57^.

**Figure 3:**
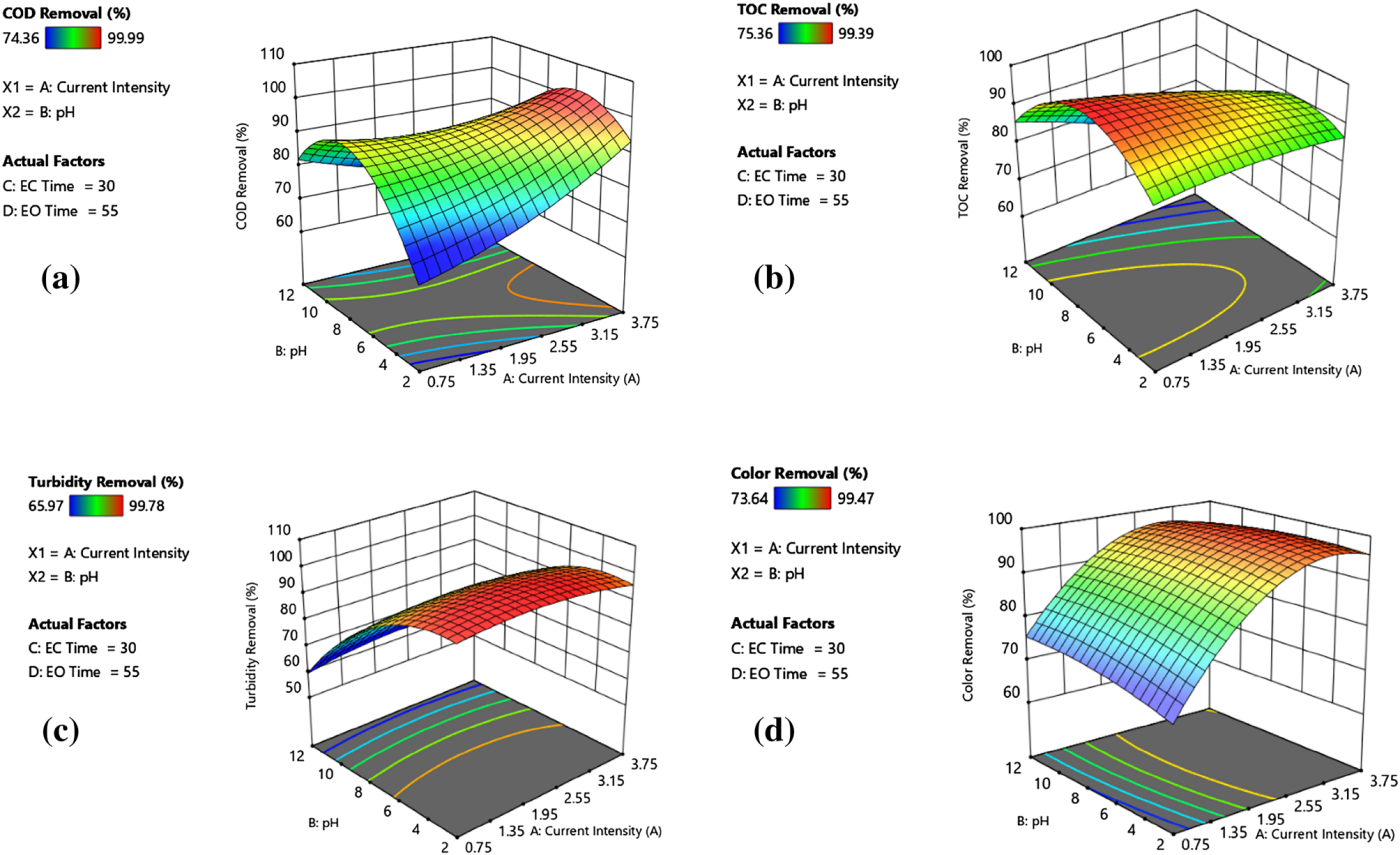
Effect of current density and initial pH on (**a**) COD removal, (**b**) TOC removal, (**c**) turbidity removal, and (**d**) colour removal.

Figure 3a–d revealed a strict relationship between initial pH and current density. While almost complete turbidity removal was achieved at an initial pH of 2, the lowest pollutant removal efficiencies were obtained at an initial pH of 12. Removal efficiencies of all pollutants were practically the same at a current density of 2.25 and an initial pH of 7. Such a high turbidity removal performance attained at an initial pH of 2 was attributed to the combined effect of charge neutralisation and sweep flocculation. This effect was clearly apparent when EC was initiated at pH 4.5. In the initial pH range of 4.5–7.0, an improvement in organic matter (COD and TOC) removal performance was evident. A similar improvement was also observed when the current density increased from 0.75 A to 3.75 A. Two mechanisms are responsible for organic matter removal; the first is adsorption/entrapment on freshly produced Al(OH)_3_(H_2_O)_3_ flocs. This mechanism was also responsible for turbidity removal in EC operation. The second mechanism is indirect oxidation in the EO reactor at acidic pH values^58,59^. EC-EO process performance tended to reduce at alkaline initial pH values due to the formation of ${\text{Al}}{\text{(OH)}}_{4}^{-}$, decrease in active sites on the surface of Al(OH)_3_(H_2_O)_3_ flocs and poor oxidative properties. Nevertheless, it could be inferred that the decolourisation rate was not significantly affected by initial pH and current density (except 0.75 and 1.5 A) as more or less the same colour removal efficiencies were attained at current intensities of 2.25 and 3.0 A.

#### Effect of electrolysis time on the process performance

**Figure 4:**
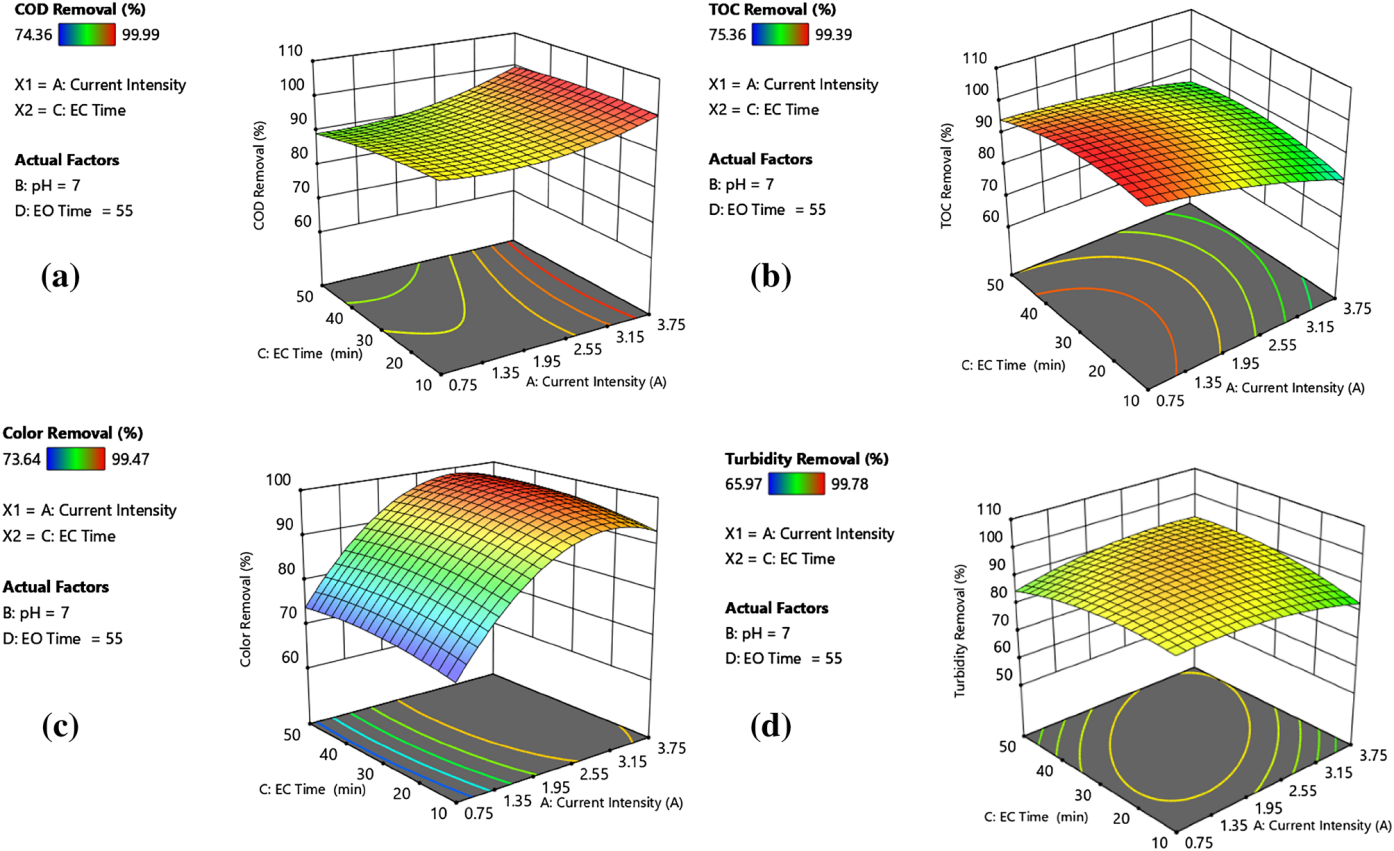
Effect of current density and EC time on (**a**) COD removal, (**b**) TOC removal, (**c**) turbidity removal, and (**d**) colour removal.

**Figure 5:**
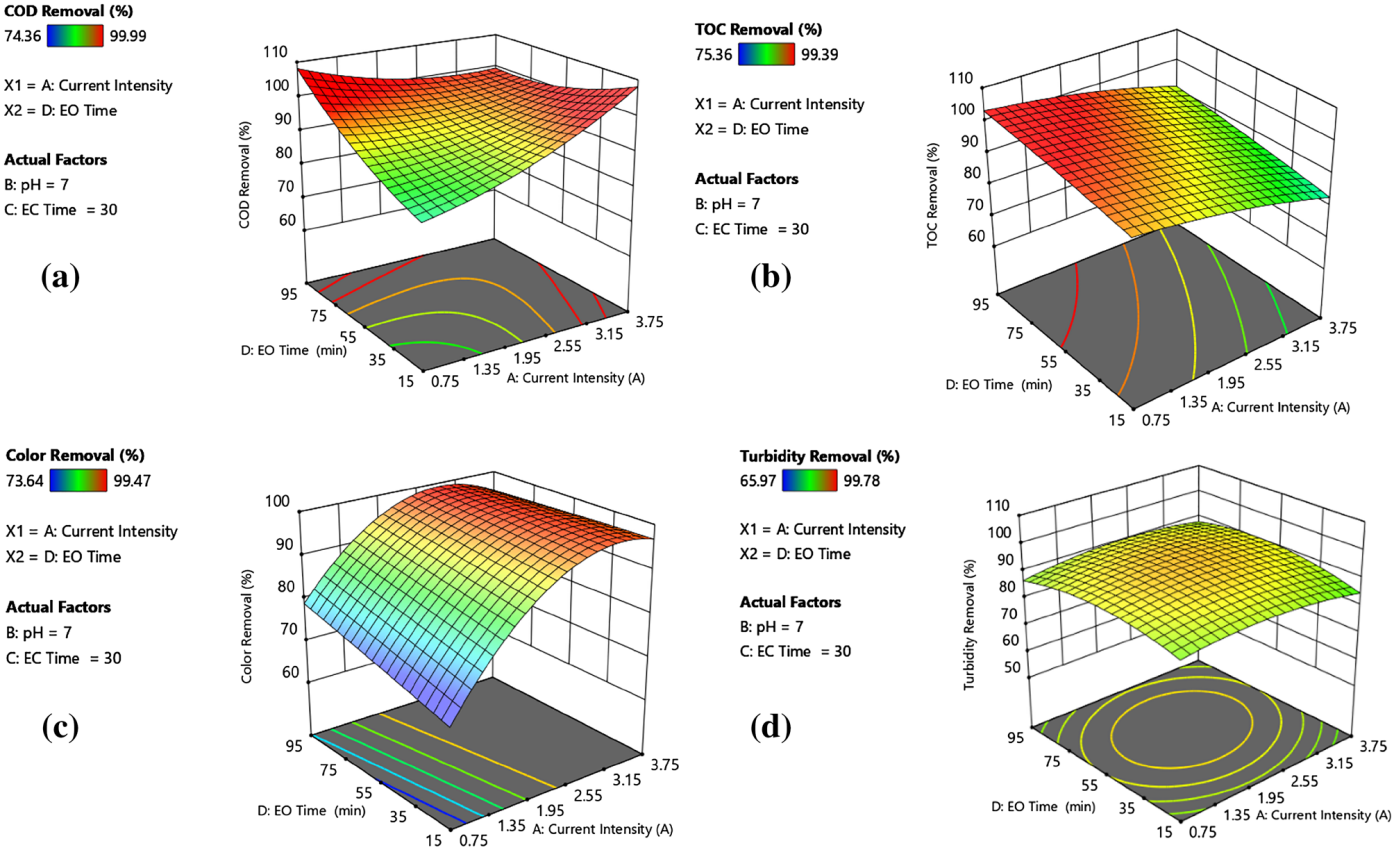
Effect of current density and EO time on (**a**) COD removal, (**b**) TOC removal, (**c**) turbidity removal, and (**d**) colour removal.

A closer inspection of data in Figs. 4 and 5 demonstrated that both EC and EO times have similar effects on process performances. Nevertheless, a slight difference between the response surfaces (cf; Figs. 4a and 5a) of COD removal performances is also evident. Although the shortest EC and EO times (10 and 15 min, respectively) yielded almost equal pollutant removal efficiencies at 2.25 A and initial pH 7, increasing the EO time from 55 to 95 min resulted in 7% additional COD removal (99.78%). Similar improvement in organic matter removal was also observed at initial pH 4.5 and current intensities of 1.5 and 3.0 A for a constant EC time of 40 min. For instance, extending the EO time from 35 to 75 min at a current density of 3.0 A and an EC time of 40 min, COD and TOC removal efficiencies increased from 91.87% and 89.81% to 97.5% and 96.4%, respectively. At the current density of 1.5 A, initial pH of 4.5 and EC time of 20, almost complete mineralisation corresponding to 99.39% TOC removal was achieved by extending EO to 75 min. Based on the data, it could be concluded that an extension in EO time promoted further degradation of organic matter via indirect oxidation. The data obtained from run 28 also confirmed this conclusion. Virtually complete COD removal (99.78%) and high TOC and colour removals (97.43% and 97.78%, respectively) were obtained by prolonging the EO time to 95 min. 20-min extension, either from 20 to 40 min or 30 to 50 min in EC time, remarkably reduced COD and TOC removals, which could be explained by a decrease in the concentration of positively charged polymeric Al species promoting charge neutralisation or in the capacity of Al hydroxide flocs as solution pH shifted to its pH_zpc_. Considering that almost the same colour and turbidity removal efficiencies were attained at the same current density (i.e. 1.5 or 3.0 A) and initial pH of 4.5 without affecting EC and EO times applied, the main responsible mechanism for removal of both pollutants is the charge neutralisation by positively charged polymeric Al hydroxo complex species or adsorption onto Al hydroxide flocs^54^.

### Comparison with the scientific literature

It should be noted that a limited number of the studies dealt with treating GW by electrochemical processes. For example, in the study of Nasr et al.^56^, the GW was treated using EC having Al electrodes, and turbidity removal increased with increasing amperage and extending reaction time up to 30 min, and increasing the EC time did not provide additional improvement in turbidity removal. Bani-Melhem et al.^60^, who used the EC method to remove COD and turbidity from solutions, reported that (i) the COD removal efficiency increased up to 96% with increasing the applied current density up to 9.36 mA/cm^2^, (ii) turbidity removal efficiency exceeded 98.5% when the applied current density was greater than 7.02 mA/cm^2^ and (iii) 15 min electrolysis time was sufficient to remove 96% COD removal. Similar effects were also noticed by Karichappan, Venkatachalam and Jeganathan^41^ during the treatment of GW by EC using stainless steel electrodes; the latter noticed that the COD, total solids (SS) and fecal coliform (FC) removal efficiencies increased with the increase of the current density up to 20 mA/cm^2^ and treatment time up to 15 min, while almost constant removal efficiencies were obtained at the higher current densities (25–30 mA/cm^2^) and EC times (15–30 min). To the best of the author’s knowledge, no information about the effect of initial pH on EC process performance for GW treatment is available in the literature. Only a few studies investigated pH change during the EC process initiated at the original pH of the GW^60,61^. Therefore, the authors are not able to compare the results presented for initial pH with literature data. The other operating parameters, such as current density and electrolysis time, exhibited similar trends in the removal of organic matter, colour and turbidity. Based on this evaluation, it could be concluded that our data were consistent with the recent scientific literature^56,60,62,63^.

### Pareto chart analysis

**Figure 6:**
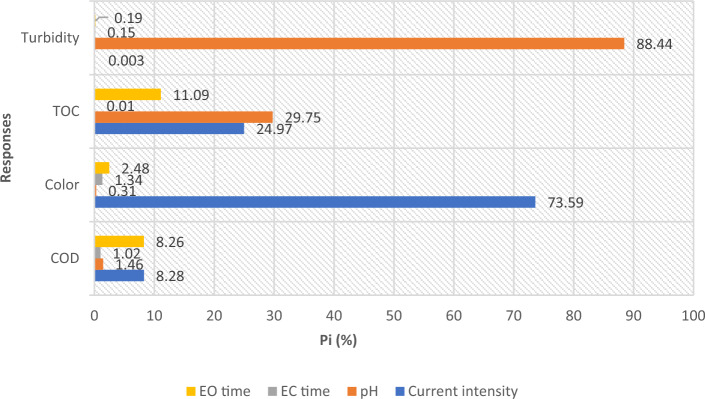
Pareto chart demonstrating the relative significance of the variables on the various responses.

As demonstrated in the ANOVA results, a precise significance level for each variable can be obtained using the sum of squares. In other words, dividing each variable sum of squares by the model sum of squares gives the order of significance of the variables on the response by arranging the variables by the ratio amount. This procedure is completed to four main variables in this process, and it is demonstrated in the Pareto chart in Fig. 6 that for COD removal, current density and EO time have a comparative effect much further than that of the two other variables, accounting for only 8.26% and 8.28, respectively. However, the current density has the main dominant effect on colour removal, with 73.59% of the independent variables’ impact. For TOC removal, current density and pH have the main effects more than EO time, and the effect of EC time on the TOC removal was negligible. Finally, pH has the main effect on turbidity removal, which overshadows other variables nearly completely by 88.44%. The Pareto chart can compare independent variables for their impact on the responses. However, as is evident from ANOVA tables, quadratic and interactive forms of the variables have significant effects in most of the responses. Equation (11) signifies the reaction direction to a high COD removal rate, which occurs in acidic pH with low current density in a long reaction time. On the other hand, Eq. (12) shows that current density and its quadratic form are the most significant variables affecting the colour removal rate further than reaction time. Equation (13) indicates that pH and its quadratic form have the most adverse effect on TOC removal, while EO time mainly contributes to the high TOC removal. Also, Eq. (14) asserts that pH and its quadratic form have the most adverse effect on turbidity removal, which means that most turbidity removal occurs at acidic pH levels. Based on these results, it could be concluded that the impact of variables and their combinations on the responses vary considerably, and it is a complex procedure to find an optimum way in order to maximise all the responses simultaneously. As a result, the RSM optimisation module is exploited in this endeavor.

### Desirability function and optimisation of experimental conditions

**Figure 7:**
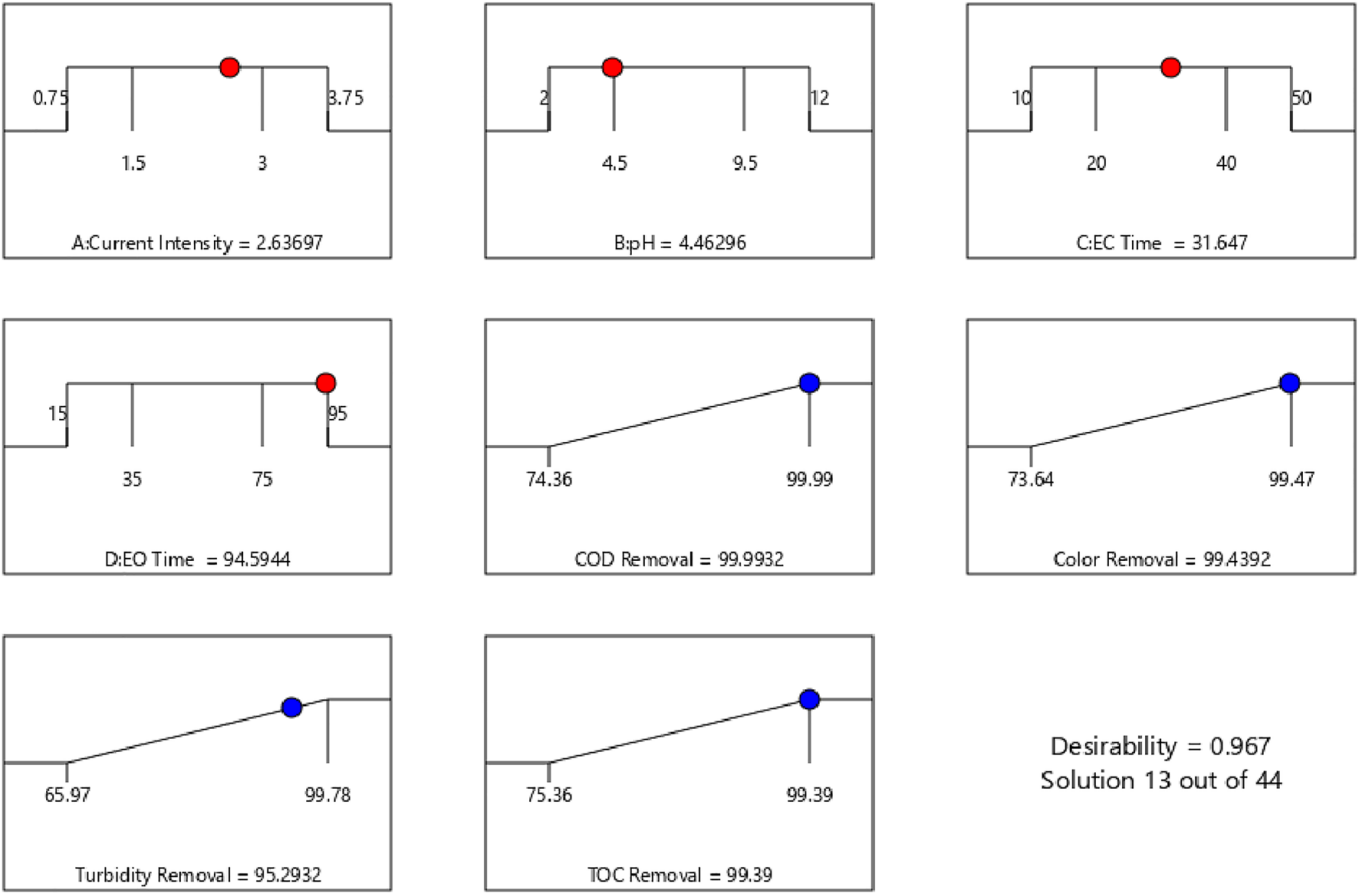
Desirability plot for optimisation (the value of variables and responses are written below each curve).

To optimise the process and reach the desired levels of responses simultaneously, the Design Expert was used to predict the optimum operational parameters. In this stage, all responses were given the same importance and weight to be maximised and the operating parameters, including current density, initial pH, EC time, and EO time, were in the range of the definitions. As it is demonstrated in Fig. 7, the software predicted 44 solutions arranged in the order of desirability from 0 to 1. One of the solutions was selected with a desirability of 0.967, which means that the probability of the prediction coming true is about 97%. The level of each factor and the corresponding responses are written below each desirability plot. The software foretells that while maintaining EO time, EC time, current density and pH at 94.5944 min, 31.647 min, 2.63697 A and 4.46296, respectively, the removal efficiencies of COD, TOC, colour, and turbidity are 99.9932%, 99.39%, 99.4392%, and 95.2932%, respectively (with a probability of 97%).

In Fig. S3, the yellow region demonstrates all analogous amounts for initial pH and current density that will result in the desired levels of the responses discussed in the desirability plot. In the yellow region, when the process was conducted at EC time = 31.67 min, and EO time = 93.28 min, current density and initial pH should be 2.6 A and 4.67 to maximise COD, colour, turbidity and TOC removals. To evaluate the optimisation accuracy, results obtained at optimal conditions showed that the COD, colour, turbidity and TOC removals were 96.1%, 97.5%, 90.9% and 98%, respectively, which were close to the predicted results (99.99%, 99.3%, 95.41%, and 99.39%, respectively). This similarity between the measured and predicted results confirms that the model can be employed to optimise the EC-EO process on larger scales.

### Operational cost

There is scarce literature on the treatment of persistent organic pollutants using integrated EC-AOPs that has investigated the cost of the operation in real wastewater. EC and EO, standalone treatment technologies, are energy-based, which consume a large amount of energy to degrade such pollutants completely. Therefore, to minimise overall energy consumption, the use of EC as a first treatment stage is recommended. Moreover, EC reduces the longer treatment time of the oxidation process, which leads to a reduction in overall treatment time as well as overall energy consumption. The EC-EO process has been found to be dependent on operational costs, which could be a major drawback, especially in large-scale industrial applications^64^. Cost determination of the EC-EO reactor was carried out by determining the cost of electricity consumption and the cost of the electrode for treating 1 m^3^ GW at the optimum conditions (I = 2.6 A, EC time = 31.67 min, EO time = 93.28 min, EC voltage = 25 V, EO voltage = 15 V, and pH = 4.67).

The operating cost (US$/kg) is calculated using Eq. (12), which involves the cost of electrical energy, electrode material, and maintenance.

12 $$OPC\left(\$/{m}^{3}\right)=\left[a\times {Q}_{energy}\right]+\left[b\times {Q}_{electrode}\right]$$

*a* and *b* are the Iranian Ministry of Energy’s pricing of 0.08 US$ per 1 kWh of energy and the Iranian market’s price of 1.95 US$ for 1 kg of Al and 2500 US$ for 1 kg of titanium, respectively. The energy consumption for each pollutant is designated by Q_energy_ (kWh/kg pollutant eliminated) (Eq. 13), whereas electrode material consumption is indicated by Q_electrode_ (kg Al/kg pollutant removed) (Eq. 14)^43^.

13 $${Q}_{energy}=\frac{U\times I\times t}{\left({C}_{0}-{C}_{t}\right)\times {V}_{R}}$$

14 $${Q}_{electrode}=\frac{{M}_{V}\times I\times t}{Z\times F\times \left({C}_{0}-{C}_{t}\right)\times {V}_{R}}$$

where *C*_*0*_ and *C*_*t*_ represent the initial and final pollutant concentrations (mg/L), respectively; *U* refers to the operating electrical potential (volt); *I* defines the applied current (A); *t* is the EC and EO time (hour); and *V*_*R*_ denotes the effluent volume (L). Electrical energy consumption (EEC) is defined as the amount of electricity consumed per 1 mg of removed pollutants. Operating costs (OPCs) were computed to determine the process’s economic viability. *M*_*V*_ is the anode’s molecular mass (Al = 26.98 g/mol in EC and Ti = 47.87 g/mol in EO); *Z* is the number of transported electrons (Z = 3 for Al and Z = 2 for Ti); and F is Faraday’s constant (96,485.33 C/mol). All chemical consumptions were ignored in the calculation, as Na_2_SO_4_ addition was unnecessary under optimal conditions.

The average OPC and energy consumption for the EC-EO process to remove COD, colour, turbidity, and TOC were calculated as 0.014$/m^3^ and 0.01kWh/kg COD removed, 0.083$/m^3^ and 0.008kWh/kg colour removed, 0.075$/m^3^ and 0.062kWh/kg turbidity removed, and 0.105$/m^3^ and 0.079kWh/kg TOC removed, respectively. Various studies described the operating cost analysis of real wastewater treatment using EC-EO by applying different electrode materials, tabulated in Table 5. As seen in the table, when scaling up the reactor, the cost of the process may change due to various expenses related to the wastewater characteristics, reactor, chemical dosage, and reagent requirements. Hence, it is essential to consider this factor during the scale-up process.

**Table 5:** Summary of the cost analysis of the EC-EO method described in the literature for real wastewater treatment.

Electrode material	Wastewater	Operating condition	Removal efficiency (%)	Cost/energy consumption	Refs.
EC (anode: Al, cathode: Gr), EO (anode: Al, cathode: Gr)	Soluble coffee production	Current density = 149.2 A/m^2^, pH = 7.98, time = 62 min	COD (89), colour (100), TOC (72)	45.28 kWh/m^3^	^64^
Anode and cathode: Gr, BP: Al	Pulp/paper mill	EC = 5 mS/cm, current = 1 A, and pH = 6	COD (87), colour (100), turbidity (100)	1.33 $/m^3^, 31.6 kWh/kg COD	^65^
Anode: TiO_2_, cathode: Al	Dairy wastewater	Current = 2 A, time = 60 min	COD (57.5) and turbidity (98.72)	17.95 €/m^3^	^66^
Anode and cathode: Gr, BP: Al	Restaurant wastewater	Current = 4 A, time = 90 min, and pH = 7	O&G (98), COD (90), BOD (86), PO_4_ (88), and turbidity (98)	1.56 US$/m^3^	^67^
Anode: Fe, cathode: CF	Domestic wastewater	Current density = 100 A/m^2^, t = 20 min and pH: 7.8	E.coli (100) and COD (97.5)	1.6 $/m^3^, 18.9 kWh/m^3^	^68^
EC (anode: Al, cathode: Ti) and EO (anode Gr, cathode: Ti)	Textile wastewater	Current density = 4.1 mA/cm^2^, pH = 4, conductivity = 3.7 mS/cm	DCOD (70)	1.47 $/m^3^	^69^
EC (anode: Al, cathode: Fe) and EO (anode: Ti/Pt, cathode: SS)	Domestic GW	Current = 2.6 A, EC time = 31.67 min, EO time = 93.28 min, and pH = 4.67	COD (96.1), colour (97.5), turbidity (90.9) and TOC (98)	0.01 kWh/kg COD; 0.008 kWh/kg colour; 0.062 kWh/kg turbidity; 0.079 kWh/kg TOC; 0.28 $/m^3^	Present study

*DCOD* chemical oxygen demand degradation, *CF* carbon felt, *Fe* iron, *PO*_4_–*P* phosphate-phosphorus, *O*&*G* oil and greases, *BOD* Five-day biochemical oxygen demand, *TiO*_2_ titanium dioxide, *BP* bipolar, *Gr* graphite.

## Conclusions

The obtained results indicated that integrating the EC and EO processes offers an applicable combination by taking full advantage of these two methods; the EC method is a rapid and effective method for wastewaters that contain suspended solids but is also an ineffective approach for the elimination of persistent dissolved organic pollutants, and EO can breakdown persistent organic pollutants completely, however, it is also a slow and energy-intensive technique for suspended solids removal. The results also indicated that the efficiency of the EC-EO treatment system in colour and turbidity removal depends on the current density and pH. The results also showed that the best operating parameters were a current density of 2.6 A, EC time of 31.67 min, EO time of 93.28 min and pH of 4.67, which resulted in removal efficiencies of 96.1% (COD), 97.5% (colour), 90.9% (turbidity), and 98% (TOC). The process's energy requirements were 0.01kWh/kg for COD, 0.008kWh/kg for colour, 0.062kWh/kg for turbidity, and 0.079kWh/kg for TOC, with an operating cost of 0.28$/m^3^. Future research should focus on developing novel and durable photo-active dimensionally stable anodes to extend the lifetime of water electrolysers, reduce operating costs, and improve overall electrochemical system performance. Moreover, to enhance the understanding of the strengths and limitations of each unit, it is recommended to evaluate individual unit performance before integrating them into a complete system. This can be achieved through analyzing the efficiency of the electrocoagulation reactor and electrooxidation cell under different operating conditions, which will offer valuable insights into their respective performance metrics.

### Supplementary Information

Supplementary Figures.

## Data Availability

The datasets generated and analysed during the current study available from the corresponding author, Milad Mousazadeh (m.milad199393@gmail.com), on reasonable request.

## References

[1] Pedro-Monzonís M, Solera A, Ferrer J, Estrela T, Paredes-Arquiola J (2015). A review of water scarcity and drought indexes in water resources planning and management. J. Hydrol..

[2] Leong JYC, Siang Oh K, Poh PE, Chong MN (2017). Prospects of hybrid rainwater-greywater decentralised system for water recycling and reuse: A review. J. Clean. Prod..

[3] Eriksson E, Auffarth K, Henze M, Ledin A (2002). Characteristics of grey wastewater. Urban Water.

[4] Al-Gheethi AA, Mohamed RMSR, Rahman MAA, Johari MR, Kassim AHM (2015). Treatment of wastewater from car washes using natural coagulation and filtration system. Proc. Soft Soil Eng. Int. Conf..

[5] Travis MJ, Wiel-Shafran A, Weisbrod N, Adar E, Gross A (2010). Greywater reuse for irrigation: Effect on soil properties. Sci. Total Environ..

[6] Friedler E, Yardeni A, Gilboa Y, Alfiya Y (2011). Disinfection of greywater effluent and regrowth potential of selected bacteria. Water Sci. Technol..

[7] Hernandez Leal L, Temmink H, Zeeman G, Buisman C (2010). Comparison of three systems for biological greywater treatment. Water.

[8] Atanasova N, Dalmau M, Comas J, Poch M, Rodriguez-Roda I, Buttiglieri G (2017). Optimized MBR for greywater reuse systems in hotel facilities. J. Environ. Manag..

[9] Abdel-Shafy HI, Al-Sulaiman AM, Mansour MS (2015). Anaerobic/aerobic treatment of greywater via UASB and MBR for unrestricted reuse. Water Sci. Technol..

[10] AlJaberi FY, Mohsen Alardhi S, Ahmed SA, Salman AD, Juzsakova T, Cretescu I, Le P-C, Chung WJ, Chang SW, Nguyen DD (2022). Can electrocoagulation technology be integrated with wastewater treatment systems to improve treatment efficiency?. Environ. Res..

[11] Miklos DB, Remy C, Jekel M, Linden KG, Drewes JE, Hübner U (2018). Evaluation of advanced oxidation processes for water and wastewater treatment–A critical review. Water Res..

[12] Zhu S, Wang C, Yip AC, Tsang DC (2015). Highly effective degradation of sodium dodecylbenzene sulphonate and synthetic greywater by Fenton-like reaction over zerovalent ironbased catalyst. Environ. Technol..

[13] Ahmadi M, Ghanbari F (2016). Optimizing COD removal from greywater by photoelectropersulfate process using Box-Behnken design: Assessment of effluent quality and electrical energy consumption. Environ. Sci. Pollut. Res..

[14] Sanchez M, Rivero MJ, Ortiz I (2010). Photocatalytic oxidation of grey water over titanium dioxide suspensions. Desalination.

[15] Chin WH, Roddick FA, Harris JL (2009). Greywater treatment by UVC/H_2_O_2_. Water Res..

[16] Teodoro A, Árpád Boncz M, Junior AM, Paulo PL (2014). Disinfection of greywater pre-treated by constructed wetlands using photo-Fenton: Influence of pH on the decay of Pseudomonas aeruginosa. J. Environ. Chem. Eng..

[17] Barzegar G, Wu J, Ghanbari F (2019). Enhanced treatment of greywater using electrocoagulation/ozonation: Investigation of process parameters. Process Saf. Environ. Prot..

[18] Daghrir R, Gherrou A, Noel I, Seyhi B (2016). Hybrid process combining electrocoagulation, electroreduction, and ozonation processes for the treatment of grey wastewater in batch mode. J. Environ. Eng..

[19] Ghanbari F, Moradi M, Eslami A, Emamjomeh MM (2014). Electrocoagulation/flotation of textile wastewater with simultaneous application of aluminum and iron as anode. Environ. Process..

[20] Arab M, Faramarz MG, Hashim K (2022). Applications of computational and statistical models for optimizing the electrochemical removal of cephalexin antibiotic from water. Water.

[21] Hashim KS, Khaddar RA, Jasim N, Shaw A, Phipps D, Kot P, Pedrola MO, Alattabi AW, Abdulredha M, Alawsh R (2019). Electrocoagulation as a green technology for phosphate removal from River water. Sep. Purif. Technol..

[22] Hakizimana JN, Gourich B, Chafi M, Stiriba Y, Vial C, Drogui P, Naja J (2017). Electrocoagulation process in water treatment: A review of electrocoagulation modeling approaches. Desalination.

[23] Song P, Yang Z, Zeng G, Yang X, Xu H, Wang L, Xu R, Xiong W, Ahmad K (2017). Electrocoagulation treatment of arsenic in wastewaters: A comprehensive review. Chem. Eng. J..

[24] Ghanbari F, Moradi M, Mohseni-Bandpei A, Gohari F, Mirtaleb Abkenar T, Aghayani E (2014). Simultaneous application of iron and aluminum anodes for nitrate removal: a comprehensive parametric study. Int. J. Environ. Sci. Technol..

[25] Garcia-Segura S, Eiband MMS, de Melo JV, Martínez-Huitle CA (2017). Electrocoagulation and advanced electrocoagulation processes: A general review about the fundamentals, emerging applications and its association with other technologies. J. Electroanal. Chem..

[26] Özyurt B, Camcıoğlu S (2018). Applications of combined electrocoagulation and electrooxidation treatment to industrial wastewaters. Wastewater Water Qual..

[27] Chen G (2004). Electrochemical technologies in wastewater treatment. Sep. Purif. Technol..

[28] Comninellis C (1994). Electrocatalysis in the electrochemical conversion/combustion of organic pollutants for waste water treatment. Electrochem. Acta.

[29] Gilpavas E, Arbeláez-Castaño P, Medina J, Acosta DA (2017). Combined electrocoagulation and electro-oxidation of industrial textile wastewater treatment in a continuous multi-stage reactor. Water Sci. Technol..

[30] Özyonar F, Korkmaz MU (2022). Sequential use of the electrocoagulation-electrooxidation processes for domestic wastewater treatment. Chemosphere.

[31] Okur MC, Akyol A, Nayir TY, Kara S, Ozturk D, Civas A (2022). Performance of Ti/RuO_2_-IrO_2_ electrodes and comparison with BDD electrodes in the treatment of textile wastewater by electro-oxidation process. Chem. Eng. Res. Des..

[32] Ghimire U, Jang M, Jung SP, Park D, Park SJ, Yu H, Oh S-E (2019). Electrochemical removal of ammonium nitrogen and COD of domestic wastewater using platinum coated titanium as an anode electrode. Energies.

[33] Barisci S, Turkay O (2016). Domestic greywater treatment by electrocoagulation using hybridelectrode combinations. J. Water Process Eng..

[34] Vakil KA, Sharma MK, Bhatia A, Kazmi AA, Sarkar S (2014). Characterization of greywater in an Indian middle-class household and investigation of physicochemical treatment using electrocoagulation. Sep. Purif. Technol..

[35] Bhagawan D, Poodari S, Golla S, Himabindu V, Vidyavathi S (2016). Treatment of the petroleum refinery wastewater using combined electrochemical methods. Desalin. Water Treat..

[36] Yılmaz Nayir T, Serdar K (2018). Container washing wastewater treatment by combined electrocoagulation–electrooxidation. Sep. Sci. Technol..

[37] Rubí-Juárez H, Linares-Hernández I, Fall C, Bilyeu B (2015). A combined electrocoagulation-electrooxidation process for carwash wastewater reclamation. Int. J. Electrochem. Sci..

[38] Bensadok K, El Hanafi N, Lapicque F (2011). Electrochemical treatment of dairy effluent using combined Al and Ti/Pt electrodes system. Desalination.

[39] Hajalifard Z, Mousazadeh M, Khademi S, Khademi N, Jamadi MH, Sillanpää M (2023). The efficacious of AOP-based processes in concert with electrocoagulation in abatement of CECs from water/wastewater. NPJ Clean Water.

[40] Bani-Melhem K, Smith E (2012). Grey water treatment by a continuous process of an electrocoagulation unit and a submerged membrane bioreactor system. Chem. Eng. J..

[41] Karichappan T, Venkatachalam S, Jeganathan PM (2014). Optimization of electrocoagulation process to treat grey wastewater in batch mode using response surface methodology. J. Environ. Health Sci. Eng..

[42] American Public Health Association (APHA), A.W.W.A.A., Water Pollution Control Federation (WPCF), Water Environment Federation (WEF). Standard Methods for the Examination of Water and Wastewater (**1915**).

[43] Olya ME, Pirkarami A (2013). Electrocoagulation for the removal of phenol and aldehyde contaminants from resin effluent. Water Sci. Technol..

[44] Hashim, K.; Al-Rifaie, J.K.; Aljaaf, A.J.; Idowu, I.; Amoako-Attah, J.; Nikitas, G. RSM (Response Surface Methodology) modelling of inter-electrodes spacing effects on phosphate removal. In Proceedings of the 2021 14th International Conference on Developments in eSystems Engineering (DeSE), 586–589 (2021).

[45] Hashim KS, Ewadh HM, Muhsin AA, Zubaidi SL, Kot P, Muradov M, Aljefery M, Al-Khaddar R (2021). Phosphate removal from water using bottom ash: Adsorption performance, coexisting anions and modelling studies. Water Sci. Technol..

[46] Abdul Rahman N, Jose Jol C, Albania Linus A, Wan Borhan WWS, Abdul Jalal NS, Baharuddin N, Samsul SNA, Abdul Mutalip NA (2023). Statistical analysis of salinity reduction in Borneo tropical brackish peat water with continuous electrocoagulation treatment system. J. Hazard. Mater. Adv..

[47] Bezerra MA, Santelli RE, Oliveira EP, Villar LS, Escaleira LA (2008). Response surface methodology (RSM) as a tool for optimization in analytical chemistry. Talanta.

[48] Jensen WA (2017). Response surface methodology: Process and product optimization using designed experiments. J. Qual. Technol..

[49] Kumari M, Gupta SK (2019). Response surface methodological (RSM) approach for optimizing the removal of trihalomethanes (THMs) and its precursor’s by surfactant modified magnetic nanoadsorbents (sMNP)—An endeavor to diminish probable cancer risk. Sci. Rep..

[50] Ansari K, Shrikhande A, Malik MA, Alahmadi AA, Alwetaishi M, Alzaed AN, Elbeltagi A (2022). Optimization and operational analysis of domestic greywater treatment by electrocoagulation filtration using response surface methodology. Sustainability.

[51] Hu CZ, Wang SQ, Sun JQ, Liu HJ, Qu JH (2016). An effective method for improving electrocoagulation process: Optimization of Al-13 polymer formation. Colloids Surf. A-Physicochem. Eng. Asp..

[52] Rebhun M, Lurie M (1993). Control of organic-matter by coagulation and floc separation. Water Sci. Technol..

[53] Kabdaşlı I, Arslan-Alaton I, Ölmez-Hancı T, Tünay O (2012). Electrocoagulation applications for industrial wastewaters: A critical review. Environ. Technol. Rev..

[54] Janpoor F, Torabian A, Khatibikamal V (2011). Treatment of laundry waste-water by electrocoagulation. J. Chem. Technol. Biotechnol..

[55] Duan JM, Gregory J (2003). Coagulation by hydrolysing metal salts. Adv. Coll. Interface Sci..

[56] Nasr M, Ateia M, Hassan K (2016). Artificial intelligence for greywater treatment using electrocoagulation process. Sep. Sci. Technol..

[57] Kabdaşlı I, Tünay O (2023). Hexavalent chromium removal from water and wastewaters by electrochemical processes: Review. Molecules.

[58] Fil BA, Boncukcuoglu R, Yilmaz AE (2023). Electro-oxidation of pistachio processing wastewater using Ti/Pt mesh-type anodes in batch system. Water Environ. J..

[59] Vlyssides A, Arapoglou D, Israilides C, Karlis P (2004). Electrochemical oxidation of three obsolete organophosphorous pesticides stocks. J. Pestic. Sci..

[60] Bani-Melhem K, Al-Shannag M, Alrousan D, Al-Kofahi S, Al-Qodah Z, Al-Kilani MR (2017). Impact of soluble COD on grey water treatment by electrocoagulation technique. Desalin. Water Treat..

[61] Bajpai M, Katoch SS, Singh M (2020). Optimization and economical study of electro-coagulation unit using CCD to treat real graywater and its reuse potential. Environ. Sci. Pollut. Res..

[62] Shakeri E, Mousazadeh M, Ahmadpari H, Kabdasli I, Jamali HA, Graca NS, Emamjomeh MM (2021). Electrocoagulation-flotation treatment followed by sedimentation of carpet cleaning wastewater: Optimization of key operating parameters via RSM-CCD. Desalin. Water Treat..

[63] Ansari K, Shrikhande A, Malik MA, Alahmadi AA, Alwetaishi M, Alzaed AN, Elbeltagi A (2022). Optimization and operational analysis of domestic greywater treatment by electrocoagulation filtration using response surface methodology. Sustainability.

[64] Ibarra-Taquez HN, GilPavas E, Blatchley ER, Gómez-García M-Á, Dobrosz-Gómez I (2017). Integrated electrocoagulation-electrooxidation process for the treatment of soluble coffee effluent: Optimization of COD degradation and operation time analysis. J. Environ. Manag..

[65] Özyurt B, Camcıoğlu Ş, Hapoglu H (2017). A consecutive electrocoagulation and electro-oxidation treatment for pulp and paper mill wastewater. Desalin. Water Treat..

[66] Turan NB (2021). The application of hybrid electrocoagulation–electrooxidation system for the treatment of dairy wastewater using different electrode connections. Sep. Sci. Technol..

[67] Daghrir R, Drogui P, Blais JF, Mercier G (2012). Hybrid process combining electrocoagulation and electro-oxidation processes for the treatment of restaurant wastewaters. J. Environ. Eng..

[68] Özyonar F, Korkmaz MU (2022). Sequential use of the electrocoagulation-electrooxidation processes for domestic wastewater treatment. Chemosphere.

[69] GilPavas E, Arbeláez-Castaño P, Medina J, Acosta DA (2017). Combined electrocoagulation and electro-oxidation of industrial textile wastewater treatment in a continuous multi-stage reactor. Water Sci. Technol..

