# Supplementary material for: Domestic greywater treatment using electrocoagulation-electrooxidation process: optimisation and experimental approaches
Source: Sci Rep. 2023 Sep 22;13:15852. doi: 10.1038/s41598-023-42831-6 (PMC10517000; doi:10.1038/s41598-023-42831-6)
Supplement: Supplementary file 1 — Supplementary Figures. [file 41598_2023_42831_MOESM1_ESM.docx]

**Optimization of domestic greywater treatment using electrocoagulation and electrochemical oxidation processes**

Milad Mousazadeh^1,2^*^†^, Nastaran Khademi^3^, Işık Kabdaşlı^4†^, Seyedahmadreza Rezaei^5^, Zeinab Hajalifard^6^, Zohreh Moosakhani^2^, Khalid Hashim^7^

^1^Social Determinants of Health Research Center, Research Institute for Prevention of Non-Communicable Diseases, Qazvin University of Medical Sciences, Qazvin, Iran

^2^Department of Environmental Health Engineering, School of Health, Qazvin University of Medical Sciences, Qazvin, Iran

^3^ Health, Safety and Environment Specialist, National Iranian Drilling Company, Ahvaz, Iran

^4^İstanbul Technical University, Civil Engineering Faculty, Environmental Engineering Department, Ayazağa Campus, 34469 Maslak, İstanbul, Turkey

^5^Department of Engineering, Faculty of Civil engineering, Persian Gulf University, Bushehr, Iran

^6^Department of Chemical Engineering, Amirkabir University of Technology, Hafez Av., Tehran, Iran

^7^Built Environment and Sustainable Technologies Research Institute (BEST), Liverpool John Moores University, Byrom Street, Liverpool L3 3AF, UK

*Corresponding author: m.milad199393@gmail.com

^†^Co-first author: Milad Mousazadeh and Işık Kabdaşlı contributed equally to this work.

(a

(b

(c

(d

**Figure S1.** Predicted vs actual diagram for removal of a) COD, b) color , c) turbidity, and d) TOC

(b

(a

**** ****

(d

(c

**Figure S2.** The normal plot of residuals for removal of a) COD, b) color , c) turbidity, and d) TOC

**Figure S3.** The overlay plot for optimal region at EC time = 31.67 min and EO time = 93.28 min.
